# Identification of neural firing patterns, frequency and temporal coding mechanisms in individual aortic baroreceptors

**DOI:** 10.3389/fncom.2015.00108

**Published:** 2015-08-26

**Authors:** Huaguang Gu, Baobao Pan

**Affiliations:** School of Aerospace Engineering and Applied Mechanics, Tongji UniversityShanghai, China

**Keywords:** neural coding, neural firing pattern, frequency coding, temporal coding, Hopf bifurcation, aortic baroreceptor, blood pressure, depolarization block

## Abstract

In rabbit depressor nerve fibers, an on-off firing pattern, period-1 firing, and integer multiple firing with quiescent state were observed as the static pressure level was increased. A bursting pattern with bursts at the systolic phase of blood pressure, continuous firing, and bursting with burst at diastolic phase and quiescent state at systolic phase were observed as the mean level of the dynamic blood pressure was increased. For both static and dynamic pressures, the firing frequency of the first two firing patterns increased and of the last firing pattern decreased due to the quiescent state. If the quiescent state is disregarded, the spike frequency becomes an increasing trend. The instantaneous spike frequency of the systolic phase bursting, continuous firing, and diastolic phase bursting can reflect the temporal process of the systolic phase, whole procedure, and diastolic phase of the dynamic blood pressure signal, respectively. With increasing the static current corresponding to pressure level, the deterministic Hodgkin-Huxley (HH) model manifests a process from a resting state first to period-1 firing via a subcritical Hopf bifurcation and then to a resting state via a supercritical Hopf bifurcation, and the firing frequency increases. The on-off firing and integer multiple firing were here identified as noise-induced firing patterns near the subcritical and supercritical Hopf bifurcation points, respectively, using the stochastic HH model. The systolic phase bursting and diastolic phase bursting were identified as pressure-induced firings near the subcritical and supercritical Hopf bifurcation points, respectively, using an HH model with a dynamic signal. The firing, spike frequency, and instantaneous spike frequency observed in the experiment were simulated and explained using HH models. The results illustrate the dynamics of different firing patterns and the frequency and temporal coding mechanisms of aortic baroreceptor.

## 1. Introduction

The encoding mechanism of sensory receptor is of fundamental importance in neuroscience (Adrian and Zotterman, 1926). The sensory receptor receives many different inputs and outputs the integrated information in the form of dynamic firing trains. In classical physiology, the output information may be encoded in the firing frequency. However, the firing frequency of temperature receptor (i.e., electroreceptor) in dogfish did not show any monotonic increasing trend as temperature increased. Rather, it first increased and then decreased (Braun et al., 1994). Different firing patterns were observed at different levels of temperature and firing patterns. It has been suggested that it may encode the temperature. An integer multiple firing by the temperature receptor was observed at low temperatures. The integer multiple firing was investigated in the nervous systems and found to be related to the stochastic resonance, indicating that noise can play important roles in neural coding (Longtin et al., 1991; Gu et al., 2001, 2011a). Like coding mechanism related to firing frequency and firing patterns, the temporal coding mechanism, which builds a relationship of temporal process between the output firing patterns and the inputs of the nervous system, has also received much attention (Brown et al., 1978; Gerstner et al., 1997; Butts et al., 2007).

Recent advances in the combination of nonlinear dynamics and neuroscience have identified various neural firing patterns, such as periodic, chaotic, and stochastic firing patterns (Yang et al., 2009; Jia et al., 2011, 2012; Jia and Gu, 2012; Gu, 2013a,b; Gu et al., 2014). Transitions between different firing patterns form a bifurcation process as some physiological parameters were changed. These included period-adding bifurcations with chaotic firing patterns, period-doubling cascade to chaos, and period-adding bifurcations with stochastic firing patterns. Some bifurcations that describe the transition from resting state to firing or vice versa, like the Hopf bifurcation and saddle-node bifurcation, have been identified (Izhikevich, 2000; Jia et al., 2011; Jia and Gu, 2012). Stochastic firing patterns were observed near the points of bifurcation (Gu et al., 2001, 2011b; Jia et al., 2011; Jia and Gu, 2012). For example, the integer multiple firing was identified as noise-induced stochastic firing near the supercritical Hopf bifurcation point (Gu et al., 2001, 2011a).

In physiological context, the neural coding mechanism of aortic baroreceptor to encode blood pressure has seen widespread investigation (Bronk and Stella, 1934; Angell James, 1971a,b; Arndt et al., 1975; Armour, 1976; Kirchheim, 1976; Brown, 1980; Chapleau et al., 1988; Seagard et al., 1990; Van Brederode et al., 1990; Cowley, 1992; Mahdi et al., 2013). Baroreceptors are sensory nerve terminals that detect blood pressure. They are the sensory components of the depressor reflex. Baroreceptors are located beneath the vascular adventitia and attach to the depressor nerve fibers. The baroreceptor reflex plays very important roles in the regulation of blood pressure. Increases in arterial blood pressure activate baroreceptors and induce afferent firings or signals propagating along depressor nerve fibers to the blood pressure regulatory center in the central nervous system (Andresen and Yang, 1992). The reflex system then reduces the blood pressure downward using the depressor reflex. How aortic baroreceptor to encode blood pressure is the beginning of the depressor reflex.

The relationship between the firing of depressor nerve fibers and the blood pressure has received a great deal of attention. The blood pressure signal *in vivo* oscillates with a period corresponding to the heart cycle. This period can be roughly divided into systolic phase, corresponding to high pressure levels, and diastolic phase corresponding to low pressure levels. It has been widely observed that the mean firing frequency increases as the mean level of blood pressure increases. A firing with burst at the systolic phase (called systolic phase bursting in the present paper) appeared at the middle level of the mean blood pressure, and a continuous firing appeared at a high mean blood pressure (Yang et al., 2006). In the experiment, an aortic baroreceptor stimulated by a static pressure that was artificially kept steady over time, a period-1 firing pattern with roughly consistent interspike intervals (ISIs) was observed (Angell James, 1971b). The firing frequency increased as the levels of both dynamic and static pressure were increased. In addition to the firing frequency and firing patterns, a temporal coding mechanism that underlies the temporal changes of pressure has been found. For example, when the pressure was artificially adjusted and the changes of the pressure obeyed a sinusoidal function, the temporal process of the instantaneous firing frequency was closely related to the sinusoidal signal (Angell James, 1971a; Arndt et al., 1975; Chapleau et al., 1988).

Recently, two irregular firing patterns, neither of which was period-1 firing, were observed in the experiment involving static pressure. One firing pattern resembled integer multiple firing (Chen et al., 2014). However, the dynamics of the two irregular firing patterns and the influence of the two irregular firing patterns on the firing frequency remain unclear. In the experiment involving dynamic blood pressure *in vivo*, except for the systolic phase bursting and the continuous firing, a bursting pattern alternating between a burst during the diastolic phase and the quiescent state during the systolic phase (called diastolic phase bursting) was observed at an extra-high level of mean blood pressure (Yang et al., 2006). The influences of diastolic phase bursting on the firing frequency and the temporal coding mechanism of the three firing patterns remain unclear. This paper addresses these questions.

In this paper, neural firing patterns, frequency, and temporal coding mechanism of the aortic baroreceptor are comprehensively investigated by the combination of biological experiment and theoretical model. The neural firing and blood pressure were simultaneously recorded in the biological experiment with static and dynamic pressures, and the relationships between the firing and blood pressure were analyzed. The deterministic HH model, stochastic HH model, and HH model using a dynamic signal to simulate blood pressure were used for the experiments. The dynamics of firing patterns, firing frequency, and temporal coding mechanisms were acquired on the basis of the bifurcation structures and combined with a mean level and temporal process of the blood pressure signals. The rest of paper is organized as follows: Section 2 presents the Materials and Methods, the results are given in Section 3, and Section 4 presents the Discussion and Conclusions.

## 2. Materials and methods

### 2.1. Experimental model

Male New Zealand white rabbits (2.0–3.0 kg) were used. All rabbits were treated in strict accordance with institutional protocols. All experiments were approved by the University Biomedical Research Ethics Committee. Surgery was performed on rabbits under anesthesia with urethane (1 g/kg, i.v.; additional doses supplemented if required during the experiment). All efforts were made to minimize suffering.

The rabbits were anesthetized and subjected to tracheal intubation with artificial ventilation to ensure that the rabbits would continue to breathe. In this paper, blood pressure was adjusted in two ways: The first was adjustment of blood pressure *in vivo* by injection of norepinephrine (NE). The mean level of blood pressure increased and instantaneous blood pressure changed over time within a cardiac period. This pattern is here called dynamic blood pressure. The second was that the inner vascular pressure was artificially controlled by isolating a segment of the rabbit aorta *in situ*. This pressure level remained unchanged over time, here called static pressure. Two different ways of adjusting pressure were performed on different rabbits. The surgical procedures for the two strategies are described as follows: (Angell James, 1971b; Brown, 1980; Sato et al., 1999; Chen et al., 2014).

#### 2.1.1. Static pressure

The right common carotid, innominate, and right subclavian arteries were exposed through midcervical and midsternal incisions. The right subclavian artery was tied proximal to the roots of the vertebral and internal thoracic arteries. Polyethylene tubing was cannulated into the right common carotid artery. Right common carotid pressure was measured via a hydraulic transducer connected to the cannulated tubing by a three-way valve. After the innominate artery was tied at its root, water sealing of the right common carotid artery was confirmed for a completely watertight cavity. In the experimental procedure, the static pressure within the sealed arterial lumen was changed directly with the change in the injected perfusion fluid volume and was measure with an MLT844 pressure transducer (ADInstruments, Australia). The right aortic depressor nerve was identified, separated, and cut off at about 2 cm in length.

#### 2.1.2. Dynamic blood pressure

Polyethylene catheters were inserted into the right common carotid artery to measure arterial pressure. Aortic pressure was measured with an ML221 pressure transducer (ADInstruments). About 2 cm of the depressor nerve was isolated. The blood pressure level was adjusted by femoral intravenous administration of NE (1:5000).

For both experiments, the distal nerve end was placed on a plastic electrode plate covered with white mineral oil and teased into divided bundles under a dissecting microscope. A single unit discharge was recorded with a platinum wire electrode. The single unit discharge signal and the pressure signal were simultaneously recorded with a PowerLab system (ADInstruments) at a sampling frequency of 10.0 kHz and monitored to make sure that the discharge signal was recorded from a single unit during the experiment. The time intervals between the maximal values of the successive spikes were recorded seriatim as an ISI series.

### 2.2. Theoretical models

#### 2.2.1. Deterministic HH model

The HH model was acquired from the squid giant axon (Hodgkin and Huxley, 1952; Hassard, 1978), and was an ideal model to describe the nerve fibers. The HH model was described as follows:

(1) $$\begin{array}{l} \frac{dV}{dt}=(g_{Na}m^{3}h(V_{Na}-V)+g_{K}n^{4}(V_{K}-V)\\ \;+g_{l}(V_{l}-V)+I)\times M \end{array}$$

(2) $$\frac{dm}{dt}=(\alpha_{m}(V)(1-m)-\beta_{m}(V)m)\times M$$

(3) $$\frac{dh}{dt}=(\alpha_{h}(V)(1-h)-\beta_{h}(V)h)\times M$$

(4) $$\frac{dn}{dt}=(\alpha_{n}(V)(1-n)-\beta_{n}(V)n)\times M$$

Here, *t* is time, and *V* is the membrane potential. In Equation (1), $g_{K}n^{4}\left(V_{K}-V\right)$, $g_{Na}m^{3}h\left(V_{Na}-V\right)$, and *g*_*l*_(*V*_*l*_ − *V*) are the current carried by K^+^, Na^+^, and the leakage current for 1 cm^2^ of the membrane, respectively. The conductance of K^+^ and Na^+^ was given by constants *g*_*K*_ and *g*_*Na*_ together with the dimensionless variables *n*, *m*, and *h*, which was consistent with Equations (4, 2, and 3). The parameters *g*_*l*_ and *V*_*l*_ represent maximum electrical conductance and equilibrium potential for leakage current channel. More detailed descriptions of the HH model have been published previously (Hodgkin and Huxley, 1952). These include the following: for example, α_*m*_ (*V*) = 0.1(25 − *V*)/(exp(25 − *V*)/10 − 1), β_*m*_ (*V*) = 4exp(−*V*/18), α_*h*_ (*V*) = 0.07exp (−*V*/20), β_*h*_ (*V*) = 1/(exp(30 − *V*)/10 + 1), α_*n*_ (*V*) = 0.01(10 − *V*)/(exp(10 − *V*)/10 − 1), and β_*n*_(*V*) = 0.125exp(−*V*/80).

In the HH model, the parameter *I* corresponds to the outward current. Variations in blood pressure cause changes in vascular stretch, which evokes the receptor potential of baroreceptors. Action potential appears when the receptor potential rises above the threshold for eliciting action potentials in the depressor nerve fibers. It is believed that changes in receptor potential over time closely resemble changes in blood pressure. In the present study, an HH model was used to simulate the depressor nerve fiber and the depolarization current *I* to simulate blood pressure.

The units of potential, current, conductance, and time are mV, μ*A*/cm^2^, mS/cm^2^, and s, respectively. The parameter values are as follows: *g*_*Na*_ = 120 mS/cm^2^, *g*_*K*_ = 36 mS/cm^2^, *g*_*l*_ = 0.3 mS/cm^2^, *V*_*Na*_ = 155 mV, *V*_*K*_ = 12 mV, and *V*_*l*_ = 10.599 mV. *M* = 1110 is a revised parameter designed to ensure that the firing frequency of the HH model approximated the experimental value.

#### 2.2.2. Stochastic HH model

Noise is inevitable in real nervous systems (Ermentrout et al., 2008). In the present study, Gaussian white noise, ξ(*t*), was used to simulate the effect of noise in the depressor nerve fiber. The statistical properties of ξ(*t*) are < ξ(*t*) > = 0 and < ξ(*t*)ξ(*t*′) > = 2*Dδ*(*t* − *t*′), where *D* is the intensity of the noise and δ(·) is the Dirac δ-function. The unit of *D* is (μA)^2^/cm^4^.

Adding ξ(*t*) to the right side of Equation (1) and the other three equations did not change the form of the stochastic HH model, which was used to simulate firing with static pressure. Equation (1) is replaced by Equation (5) as follows:

(5) $$\begin{array}{l} \frac{dV}{dt}=(g_{Na}m^{3}h(V_{Na}-V)+g_{K}n^{4}(V_{K}-V)\\ \;+g_{l}(V_{l}-V)+I+\xi (t))\times M \end{array}$$

Here, *I* was changed to simulate the changes of static pressure in the experiment.

#### 2.2.3. HH model with a signal

The dynamic blood pressure signal has two important factors: oscillation and the mean level of the pressure. In the present paper, a signal with zero mean, *BP*(*t*), was used to simulate the oscillation, and a constant, *I*, was used to simulate the mean level of blood pressure. These were introduced into Equation (1) to produce Equation (6) given as follows:

(6) $$\begin{array}{l} \frac{dV}{dt}=(g_{Na}m^{3}h(V_{Na}-V)+g_{K}n^{4}(V_{K}-V)\\ \;+g_{l}(V_{l}-V)+I+BP(t))\times M \end{array}$$

Equation (6) combined with Equations (2–4) forms an HH model with a dynamic signal to simulate the dynamic blood pressure. *I* was selected to simulate the mean blood pressure. *BP*(*t*) was assigned to an experimental recording of blood pressure signal with a mean of zero.

#### 2.2.4. Integration

The deterministic HH model, stochastic HH model, and HH model with a dynamic signal were solved using the Euler integration method with time iterations of 0.000001 s.

### 2.3. Time series analysis

In the present paper, autocorrelation function was used to identify deterministic or stochastic dynamics of ISI series of the non-periodic firing patterns. The autocorrelation function of a time series *ISI*(*i*) (*i* = 1, 2, 3, …, L) was calculated as follows: $\rho \left(\tau \right)=\frac{<\bar{ISI\left(i\right)}\bar{ISI\left(i+\tau \right)}\;>}{<{\bar{ISI\left(i\right)}}^{2}>}$, where $\bar{ISI\left(i\right)}=ISI\left(i\right)-<ISI\left(i\right)>$, < > is the average over time, and τ(τ < L) is the lag time. ρ(τ) = 1 when τ = 0. Theoretically, a time series is stochastic if ρ(τ) = 0 for all τ > 0, and it is chaotic or deterministic if ρ(τ)≠ 0 for at least one τ > 0. In practice, −0.05 < ρ(τ) < 0.05 means ρ(τ) ≈ 0.

## 3. Results

### 3.1. Experimental results

#### 3.1.1. Results of the experiment with static pressure

Experiments were performed on 26 nerve fibers from 12 rabbits. Different individual fibers manifested similar changes in firing patterns as static pressure increased. The detailed dynamics were introduced as follows.

**Figure 1:**
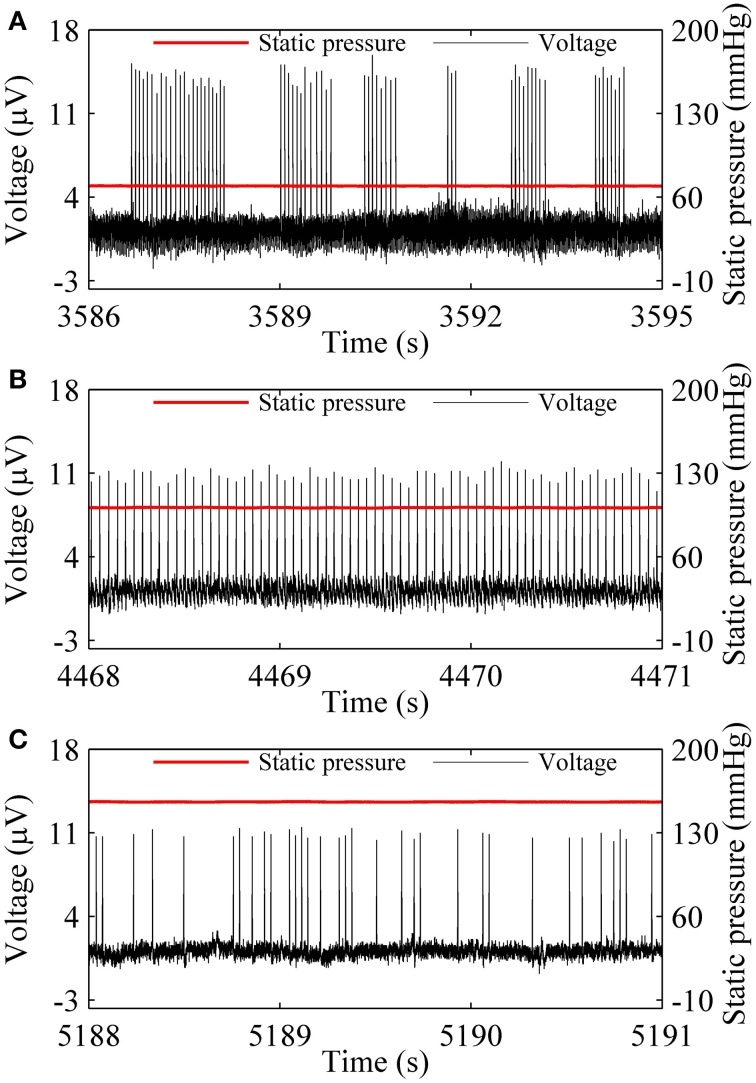
**Spike trains of firing pattern (black line) at different level of static pressure (red line) in a nerve fiber. (A)** On-off firing at 70.77 mmHg; **(B)** period-1 firing at 101.11 mmHg; **(C)** Integer multiple firing at 155.91 mmHg.

Three firing patterns, on-off firing, period-1 firing, and integer multiple firing were recorded at 70.77, 101.11, and 159.91 mmHg from a nerve fiber, as shown in Figures 1A–C, respectively. The on-off firing pattern appeared as alternation between burst with multiple spikes in a cluster and quiescent state over time. The spikes within bursts exhibited nearly equal ISI values. The durations of both burst and quiescent state showed considerable variation. The period-1 firing exhibited nearly equal ISI values. For the integer multiple firing pattern, there was a basic ISI. Other ISI values were integer multiples of the basic ISI. The ISI longer than the basic ISI corresponds to quiescent state. The integer multiple firing pattern was found to be similar to those observed in neural pacemakers and dogfish in previous studies (Braun et al., 1994; Gu et al., 2001).

**Figure 2:**
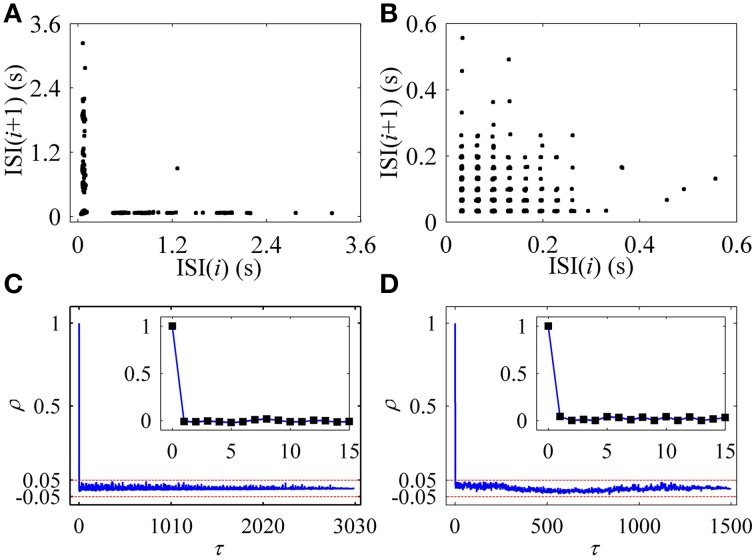
**Characteristics of firing patterns**. The first return map of ISI series: **(A)** on-off firing; **(B)** integer multiple firing pattern. Autocorrelation function of ISI series: **(C)** on-off firing; **(D)** integer multiple firing.

In the first return map of ISI series [ISI(*i*)−ISI(*i*+1), *i* is the sequential number of ISI], the on-off firing exhibited two lines paralleling to two coordinates, as shown in Figure 2A, and the integer multiple firing manifested a lattice-like structure, as depicted in Figure 2B. ρ(τ) of both on-off firing and integer multiple firing were nearly equal to 0 (−0.05 < ρ(τ) < 0.05) for all τ > 0, as shown in Figures 2C,D, respectively. The results indicate that the two firing patterns were stochastic.

**Figure 3:**
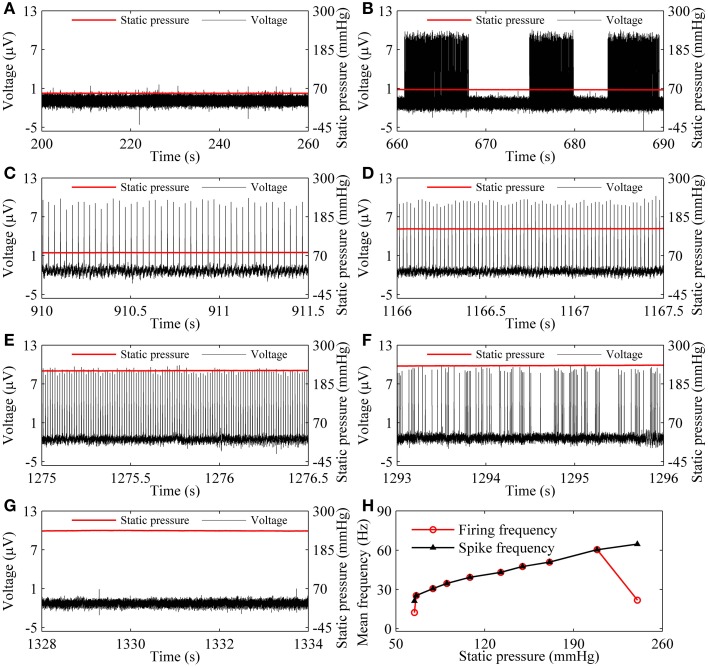
**The firing patterns (black line) observed from a depressor nerve fiber subjected to increasing levels of static pressure (red line) and firing frequency. (A)** Resting state at 55.64 mmHg; **(B)** on-off firing at 66.17 mmHg. Period-1 firing pattern: **(C)** 79.14 mmHg; **(D)** 149.83 mmHg; **(E)** 224.92 mmHg; **(F)** integer multiple firing at 240.31 mmHg; **(G)** resting state at 241.36 mmHg; **(H)** changes of mean firing frequency (red line) and mean spike frequency (black line) exclusive quiescent state with increasing static pressure level.

As the static pressure level increased, the resting state (55.64 mmHg, Figure 3A) changed to the on-off firing (66.17 mmHg, Figure 3B), to period-1 firing [79.14 mmHg (Figure 3C), 149.83 mmHg (Figure 3D), and 224.92 mmHg (Figure 3E)], to integer multiple firing (240.31 mmHg, Figure 3F), and to resting state (241.36 mmHg, Figure 3G), was observed in a depressor nerve fiber. The spike trains of the firing and the static pressure are indicated by the black and red lines, respectively.

For the nerve fiber of Figures 3A–G, as pressure was increased, the mean firing frequency of the on-off firing and period-1 firing also increased, but the integer multiple firing decreased, as shown by the red dotted line in Figure 3H. The first and last red dots correspond to the on-off firing and the integer multiple firing, respectively, and other dots to period-1 firing. The decrease in mean firing frequency was caused by the quiescent state of the integer multiple firing. If the quiescent state of the integer multiple firing and of the on-off firing pattern was disregarded, the mean spike frequency without regard to the quiescent state took on an increasing trend, as indicated by the black line with triangles in Figure 3H.

#### 3.1.2. Results of experiment with dynamic blood pressure

**Figure 4:**
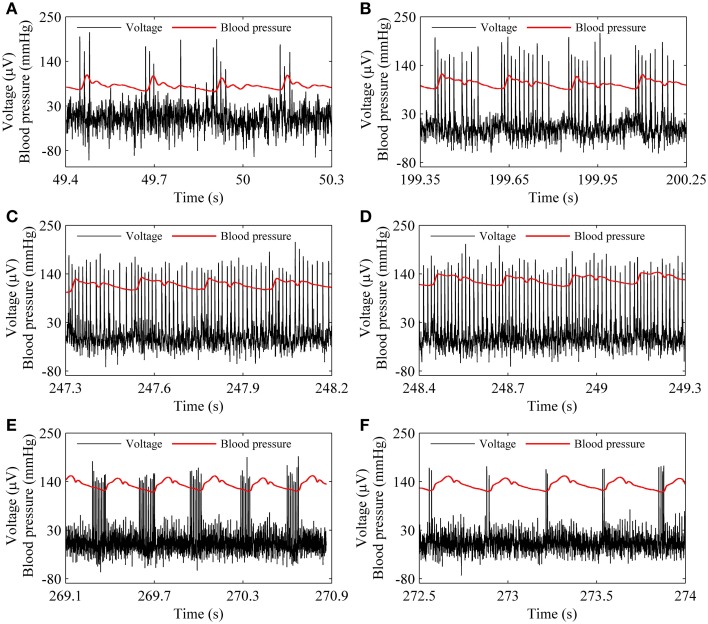
**The spike trains of firing pattern (black line) and dynamic blood pressure (red line) at different levels of mean blood pressure as observed in a depressor nerve fiber**. Systolic phase bursting at the following mean levels: **(A)** 79.55 mmHg; **(B)** 99.23 mmHg. Continuous firing at mean level: **(C)** 115.63 mmHg; **(D)** 126.58 mmHg. Diastolic phase bursting at mean level: **(E)** 131.74 mmHg; **(F)** 132.52 mmHg.

The spike trains of firing pattern (black line) and blood pressure (red line) during increases in mean level of blood pressure were simultaneously recorded in a depressor nerve fiber, as shown in Figure 4. The firing patterns were the systolic phase bursting with several spikes per burst (79.55 mmHg; Figure 4A) and with many spikes per burst (99.23 mmHg, Figure 4B), continuous firing (115.63 mmHg and 126.58 mmHg; Figures 4C,D), and diastolic phase bursting with multiple spikes per burst (131.74 mmHg; Figure 4E) and with several spikes per burst (132.52 mmHg; Figure 4F). Diastolic phase bursting appeared at an extra-high mean blood pressure, as shown in Figures 4E,F.

**Figure 5:**
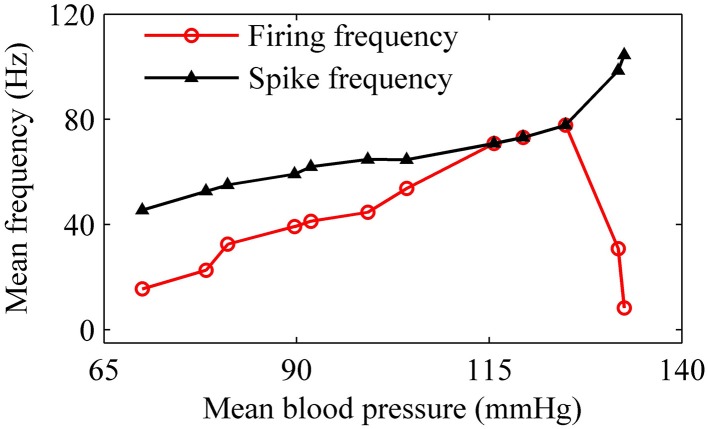
**The mean firing frequency (red line with dots) and mean spike frequency (black line with triangles) exclusive quiescent state with increasing level of mean blood pressure**.

For the nerve fiber of Figure 4 with increasing level of mean blood pressure, the mean firing frequency of the systolic phase bursting and continuous firing increased, and of the diastolic phase bursting decreased, as shown by red line with cycles in Figure 5. The decrease was induced by the quiescent state at systolic phase of the diastolic phase bursting. If the quiescent state of systolic and diastolic phase bursting patterns was ignored, the mean spike frequency exclusive the quiescent state also increased as the mean blood pressure increased, as depicted by black lines with triangles in Figure 5. The first seven, the next three, and the last two cycles (triangles) correspond to systolic phase bursting, continuous firing, and diastolic phase bursting, respectively.

**Figure 6:**
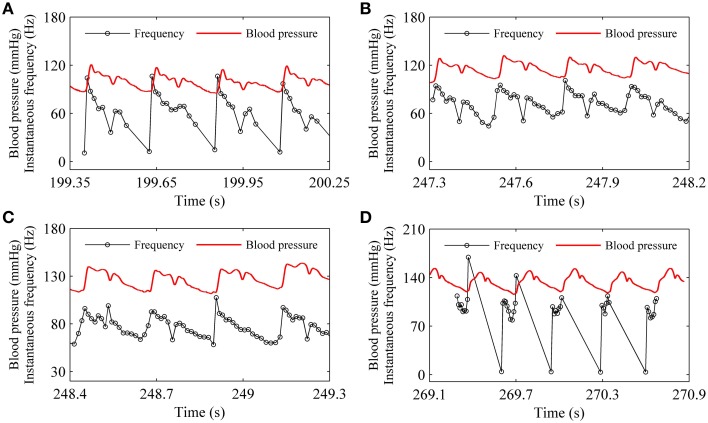
**Relationship between temporal processes between the instantaneous spike frequency (black line with dots) and blood pressure (red line). (A)** Systolic phase bursting; **(B)** continuous firing; **(C)** continuous firing; **(D)** diastolic phase bursting.

For the systolic phase bursting pattern shown in Figure 4B, the temporal process of the instantaneous spike frequency (black line with cycles) was similar to the blood pressure (red line) of the systolic phase to a certain extent, as shown in Figure 6A. For the continuous firing pattern (Figures 6B,C), the temporal process of the instantaneous firing frequency resembled the blood pressure closely. The temporal process of the instantaneous spike frequency of the diastolic phase bursting was similar to the blood pressure during the diastolic phase, as shown in Figure 6D. Different firing patterns were found to reflect different phases of blood pressure. It was because that the period (several 100 ms) of the blood pressure was much slower than the ISI value (tens of milliseconds) of period-1 firing in the depressor nerve fibers.

The experiments were performed on 32 depressor nerve fibers from 20 rabbits. Most fibers only exhibited systolic phase bursting and continuous firing. This was because the mean blood pressure was not high enough. Five nerve fibers manifested diastolic phase bursting. The changes in firing patterns showed increasing level of mean blood pressure were similar to those described in Figures 4, 5.

### 3.2. Results of the simulation

#### 3.2.1. Simulation results of deterministic HH model

**Figure 7:**
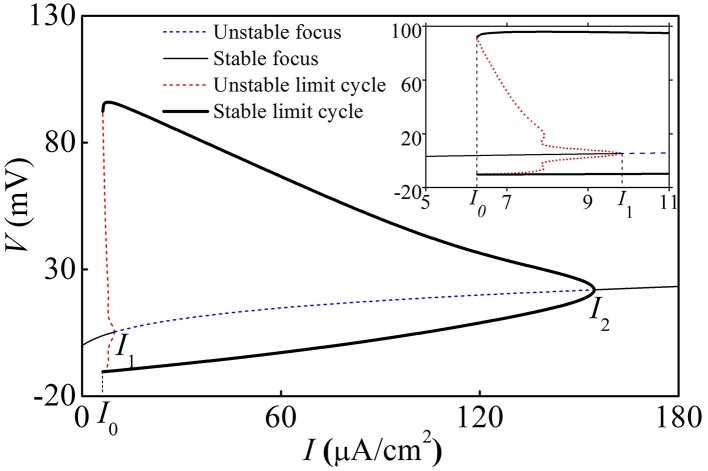
**Bifurcation structures of the deterministic HH model**. Insert is the enlargement of figure when *I*_0_ < *I* < *I*_1_. *I*_0_, *I*_1_, and *I*_2_ are a fold bifurcation point of limit cycle, a subcritical Hopf bifurcation point, and a supercritical Hopf bifurcation point.

With increasing *I*, the deterministic HH model exhibits a fold bifurcation of limit cycle at *I* = *I*_0_ ≈ 6.27 μ*A*/cm^2^, a subcritical Hopf bifurcation at *I* = *I*_1_ ≈ 8.92 μ*A*/cm^2^, and a supercritical Hopf bifurcation at *I* = *I*_2_ ≈ 154.69 μ*A*/cm^2^, respectively, as shown in Figure 7. The upper (lower) black bold line, the thin black solid line, the upper (lower) dashed red line, and the dashed blue line correspond to maximal (minimal) values of stable limit cycle, stable focus, maximal (minimal) value of unstable limit cycle, and unstable focus, respectively.

When *I* < *I*_0_ or *I* > *I*_2_, the HH model exhibited a stable equilibrium point (solid thin black line) and the behavior was quiescent. When *I*_1_ < *I* < *I*_2_, the behavior involved period-1 firing corresponding to the stable limit cycle. When *I*_0_ < *I* < *I*_1_, the quiescent state corresponding to a stable equilibrium and period-1 firing corresponding to the stable limit cycle coexist, separated by the unstable limit cycle.

**Figure 8:**
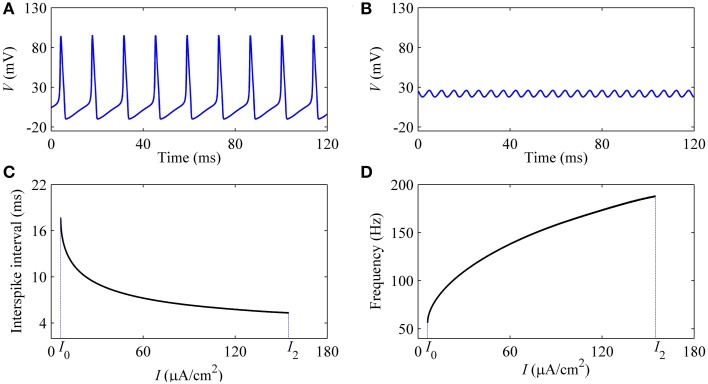
**Characteristics of firing simulated in the deterministic HH model**. The period-1 firing: **(A)** *I* = 9 μ*A*/cm^2^; **(B)** *I* = 154 μ*A*/cm^2^. Changes of firing with respect to *I*: **(C)** ISI; **(D)** firing frequency.

With increasing *I*, the oscillation amplitude of membrane potential decreases, as shown by the upper (lower) black bold line in Figure 7. For example, the amplitude of firing when *I* = 9 μ*A*/cm^2^ (Figure 8A), which is near *I*_1_, is much larger than that of *I* = 150 μ*A*/cm^2^ (Figure 8B), which is near *I*_2_. With increasing *I*, the ISI decreases, as shown in Figure 8C, correspondingly, the firing frequency increases, as shown in Figure 8D, which was similar to traditional viewpoint of frequency coding mechanism in which firing frequency showed a positive correlation to the stimulus strength.

#### 3.2.2. Simulation results of the stochastic HH model

**Figure 9:**
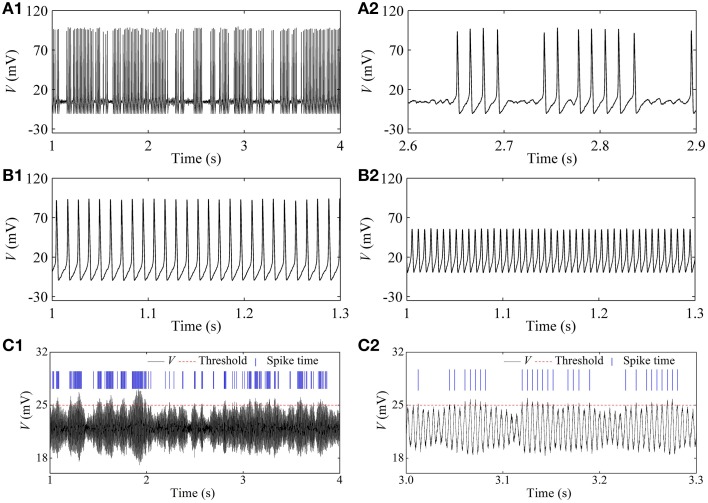
**Firing patterns at different *I*-values in the stochastic HH model [*D* = 1 (μA)^2^/cm^4^]. (A1)** On-off firing with *I* = 7.6 μ*A*/cm^2^; **(A2)** enlargement of **(A1)**; **(B1)** period-1 firing with *I* = 15 μ*A*/cm^2^; **(B2)** period-1 firing with *I* = 80 μ*A*/cm^2^; **(C1)** integer multiple firing with *I* = 154 μ*A*/cm^2^; **(C2)** enlargement of **(C1)**.

In the stochastic HH model, the on-off firing and the integer multiple firing is simulated near the subcritical and supercritical Hopf bifurcation points, respectively, within a large range of noise intensity. Representative results when *D* = 1 (μA)^2^/cm^4^ are given in Figure 9. A resting state (*I* = 2 μ*A*/cm^2^, not shown here), on-off firing (Figure 9A1, *I* = 7.6 μ*A*/cm^2^), period-1 firing pattern (Figure 9B1 with *I* = 15 μ*A*/cm^2^), and Figure 9B2 with *I* = 80 μ*A*/cm^2^), an irregular firing pattern (Figure 9C1, *I* = 154 μ*A*/cm^2^), and a resting state (*I* = 165 μ*A*/cm^2^, not shown here) are simulated.

For the irregular firing shown in Figure 9C1, only oscillation with large amplitude can trigger an action potential which corresponds to the spike recorded in the experiment. In the present study, a threshold of 25 mV (red dashed horizontal line) was set to identify action potential. The peaks of action potential are here labeled by blue vertical short line segments and the intervals between two continual vertical lines were regarded as ISIs. If a threshold different from 25 mV is chosen, the results of ISI series were similar to that of threshold of 25 mV. The ISI series was the same as that of the integer multiple firing.

**Figure 10:**
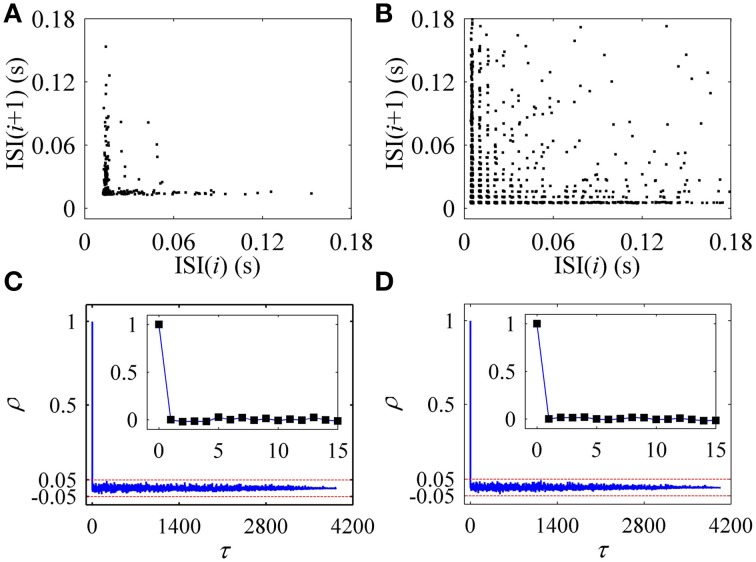
**The first return map and autocorrelation function of ISI series of firing patterns simulated in the stochastic HH model**. The first return map: **(A)** on-off firing pattern; **(B)** integer multiple firing pattern corresponding to Figure 9C1. Autocorrelation function: **(C)** on-off firing pattern; **(D)** integer multiple firing pattern corresponding to Figure 9C1.

The first return map of ISI series of on-off firing and integer multiple firing is shown in Figures 10A,B, respectively, similar to those of the experiments (Figures 2A,B). ρ(τ) of ISI series of on-off firing and integer multiple firing nearly equals 0 (−0.05 < ρ(τ) < 0.05) for all τ > 0, as shown in Figures 10C,D, showing that both firing patterns were stochastic. The results were similar to those of the experiments shown in Figures 2C,D. The on-off firing and integer multiple firing was noise-induced stochastic firings near the subcritical and supercritical Hopf bifurcation points, respectively. The results of integer multiple firing were found to be consistent with those of a previous study (Gu et al., 2001).

Oscillations with small amplitude corresponding to quiescent state in the integer multiple firing shown in Figure 9C with *I* = 154 μ*A*/cm^2^, which was similar to the supercritical Hopf bifurcation point at *I*_2_ ≈ 154.69 μ*A*/cm^2^. This was caused by noise-induced behavior near the neighborhood of the stable focus near the supercritical Hopf bifurcation point.

**Figure 11:**
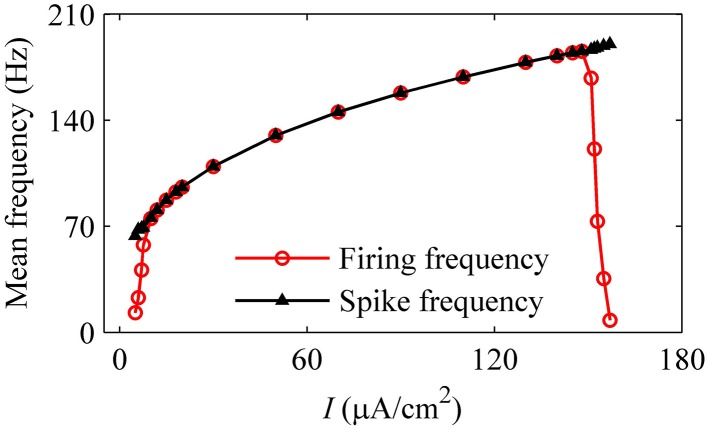
**Mean firing frequency (red line with cycles) and mean spike frequency (black line with triangles) in the stochastic HH model**.

With increasing *I*, the mean firing frequency (black line with triangles) increases for on-off firing and period-1 firing and decreases for the integer multiple firing, and mean spike frequency exclusive the quiescent state (red line with cycles) increases, as shown in Figure 11. All these results matched those of the experiment closely (Figure 3H). The decrease in the firing frequency was found to be induced by quiescent state, which is induced by noise.

#### 3.2.3. Speculation of the firing of HH model with a dynamic signal

**Figure 12:**
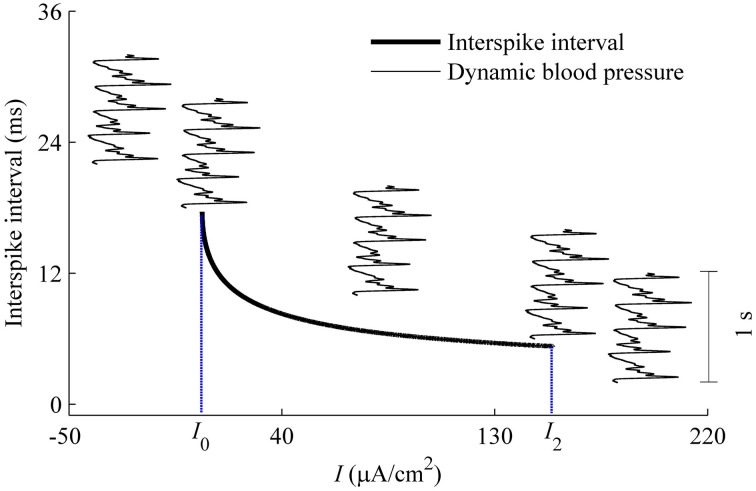
**Different firing patterns in the HH model with a dynamic signal to simulate the blood pressure**. The decreasing bold line is the (*I*, ISI) curve of the deterministic HH model. Five thin oscillating curves correspond to *I* + *BP*(*t*) with 5 different *I*-values. The five positions between *I* + *BP*(*t*) and (*I*, ISI) curve present two quiescent states and 3 dynamic firing patterns. The vertical line segment of 1 s on the right was used as time scale to measure *I* + *BP*(*t*).

As described using Equation (6), dynamic blood pressure is composed of *I* + *BP*(*t*). We plot the changes of ISI of the deterministic HH model with respect to *I*, i.e., the curve of (*I*, ISI), and a dynamic blood pressure *I* + *BP*(*t*) in Figure 12. There were five positions between (*I*, ISI) curve (bold monotone decreasing line) and *BP*(*t*) + *I* (5 thin oscillating curves) as *I* is adjusted. From left to right, corresponding to the increases in *I*, the five positions are *I*_1_ larger than the maximal value of *BP*(*t*) + *I* (position 1), *I*_1_ lying between the maximal and minimal values of *I* + *BP*(*t*) (position 2), *I* + *BP*(*t*) lay between *I*_1_ and *I*_2_ (position 3), *I*_2_ lay between maximal and minimal values of *I* + *BP*(*t*) (position 4), and *I*_2_ was below the minimal values of *I* + *BP*(*t*) (position 5). *I* + *BP*(*t*), which is much lower than the ISI values measured by the scale of the left *y*-coordinate. It was here measured using a time scale labeled by a vertical line segment of 1 s located on the right. For the 5 positions, two resting states and three dynamic firing patterns can be reasonably speculated. Positions 1 and 5 correspond to resting states. Positions 2–4 present systolic phase bursting, continuous firing, and diastolic phase bursting, respectively.

#### 3.2.4. Simulation results of an HH model with a dynamic signal

**Figure 13:**
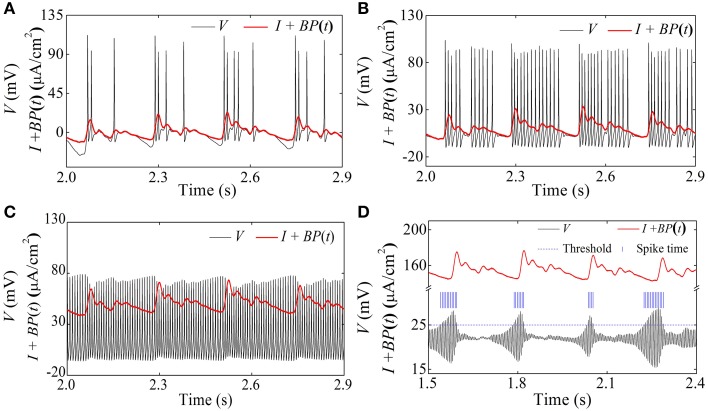
**Spike trains of firing patterns (black lines) and the blood pressure (red lines) in the HH model with a dynamic signal. (A)** Systolic phase bursting with several spikes per burst when *I* = 0 μ*A*/cm^2^; **(B)** systolic phase bursting with many spikes per burst when *I* = 10 μ*A*/cm^2^; **(C)** continuous firing when *I* = 50 μ*A*/cm^2^; **(D)** diastolic phase bursting when *I* = 154 μ*A*/cm^2^.

When *I* = 0 μ*A*/cm^2^, *I* = 10 μ*A*/cm^2^, and *I* = 50 μ*A*/cm^2^, the HH model exhibits systolic phase bursting with several spikes per burst, with many spikes per burst, and continuous firing pattern, respectively, as shown in Figures 13A–C. The firing patterns are similar to those of experiment shown in Figures 4A–D.

When *I* = 154 μ*A*/cm^2^, an oscillation with large amplitude at diastolic phase and small amplitude at systolic phase appears, as shown in Figure 11D. If the threshold to identify an action potential is chosen as 25 mV (blue dashed horizontal line), the positions of the identified action potential or spike are marked with blue vertical line segments, as depicted in Figure 13D. The firing pattern was a diastolic phase bursting pattern, which matched the experimental values closely (Figures 4E,F). The oscillation with small amplitude appeared at the systolic phase and was caused by the systolic-pressure-induced behavior near the stable focus across the supercritical Hopf bifurcation point *I*_2_.

**Figure 14:**
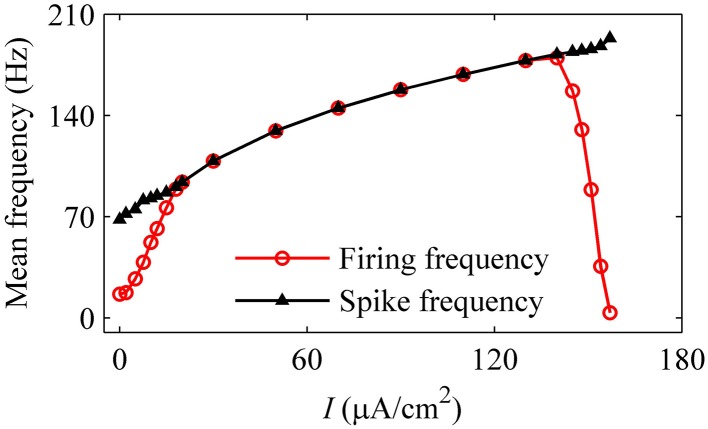
**Changes in the mean firing frequency (red line with cycles) and mean spike frequency exclusive quiescent state (black line with triangles) with respect to mean level of blood pressure in the HH model with a dynamic signal**.

As the level of mean blood pressure *I* is increased, the mean firing frequency (red line with cycles) of systolic phase bursting and continuous firing increases and decreases for the diastolic phase bursting, and mean spike frequency (black line with triangles) exhibits an increasing trend, as shown in Figure 14. All these were similar to those of experiments (Figure 5). The decrease in the firing frequency was induced by the systolic pressure-induced quiescent state during the systolic phase.

**Figure 15:**
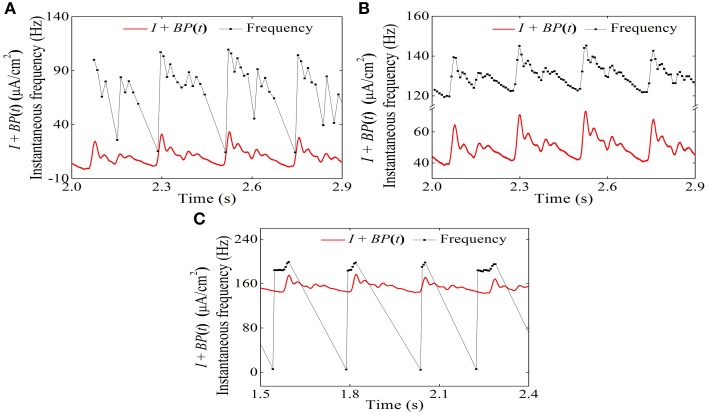
**Temporal process relationship between the instantaneous firing frequency and the blood pressure in the HH model with a dynamic signal. (A)** Systolic phase bursting; **(B)** continuous firing; **(C)** diastolic phase bursting.

The temporal process of the instantaneous firing frequency (black line) and blood pressure (red line) of the systolic phase bursting pattern, continuous firing, and diastolic phase bursting are shown in Figures 15A–C, respectively. The instantaneous firing frequency of systolic (diastolic) phase bursting pattern was found to reflect the blood pressure at systolic (diastolic) phase to a certain extent and that of continuous firing was found to reflect the blood pressure throughout a whole period. All these results were consistent with experimental values (Figure 6).

## 4. Discussion

The experimental results combined with the simulation results, the dynamics of the firing patterns, frequency and temporal coding mechanisms of the aortic baroreceptor were identified. The bifurcation structure and the increase in ISI of the deterministic HH model with increasing *I* were found to play important roles in the identification of firing patterns and neural coding mechanisms. The behaviors of the nervous system with static or dynamic pressure are from stochastic disturbance induced by noise or dynamic disturbance of the blood pressure to the bifurcation structure of the deterministic HH model. Based on such a theoretical framework, the on-off firing (Paydarfar et al., 2006; Wang, 2010) and integer multiple firing are identified as noise-induced stochastic firing patterns near subcritical and supercritical Hopf bifurcations, respectively, and the systolic phase bursting and diastolic phase bursting were found to be dynamic blood pressure-induced firing patterns near the subcritical and supercritical Hopf bifurcations, respectively, and the frequency and temporal coding mechanisms observed in the experiment were also well-explained.

When the quiescent state was disregarded, the mean spike frequency was able to reflect the blood pressure level but the mean firing frequency was not. The appearance of the integer multiple firing (Chen et al., 2014) or the diastolic phase bursting (Yang et al., 2006) at a high level pressure induced decreases in the mean firing frequency. These decreases showed that the traditional mean frequency coding mechanism loses efficacy when the blood pressure was extra-high. The decrease in firing frequency was induced by the quiescent state in the integer multiple firing and diastolic phase bursting patterns. For the integer multiple firing, noise forced the membrane potential to reach the quiescent state corresponding the stable focus at high *I* at some time to form the longer ISIs. For diastolic phase bursting, blood pressure at systolic phase forced the membrane potential to reach a quiescent state at extra-high values of *I*. If the quiescent states were disregarded, the mean frequency of the remaining spikes was found to be mainly related to the firing frequency of the deterministic HH model, showing an increasing trend with increasing pressure level. The mean spike frequency provided a modified firing frequency coding mechanism.

The temporal process of the instantaneous firing frequency was found to reflect the temporal process of the blood pressure for the bursting with long burst or the continuous firing pattern. The systolic phase bursting, continual firing, and diastolic phase bursting were found to reflect temporal processes within the systolic phase, whole period, and diastolic phase of the blood pressure. This was because the dynamic blood pressure exerted a much lower signal than the spikes of the deterministic HH model. The ISIs between two spikes, which were related to the instantaneous spike frequency, were found to reflect the pressure level between two spikes to a high extent. In this way, the instantaneous firing frequency can reflect the temporal process of the blood pressure.

In addition, the quiescent state of the integer multiple firing and of the diastolic phase bursting may be the behaviors of depolarization block (Chiodo and Bunney, 1983, Bianchi et al., 2012), which has been reported to describe termination of firing acquired by intracellular recording during depolarization. As simulated in many theoretical models, such as the HH model, FitzHugh-Nagumo model, Hindmarsh-Rose model, and Morris-Lecar model, there exists a bifurcation point from firing to resting state at a high level of depolarization current. A neuron is capable of depolarization block when it works near such a bifurcation point. Because it is very difficult to perform intracellular recording of membrane potential of the baroreceptor and of the initial axon segment of the depressor nerve fiber, it was not possible to build a direct relationship between the two firing patterns (the integer multiple firing and the diastolic phase bursting) acquired by extracellular recording and depolarization block, but the parameter range and the behaviors of the two firing patterns closely match those of the depolarization block.

In conclusion, the dynamics of neural firing patterns and the neural mechanism to encode blood pressure including mean level and temporal process of individual aortic baroreceptors were found with the help of combination of nonlinear dynamics and neuroscience. The results show that the bifurcation structures in physiological parameter space can help identify dynamics of neural firing patterns and neural coding mechanisms of the sensory receptors.

### Conflict of interest statement

The authors declare that the research was conducted in the absence of any commercial or financial relationships that could be construed as a potential conflict of interest.
